# Human mitochondrial disease-like symptoms caused by a reduced tRNA aminoacylation activity in flies

**DOI:** 10.1093/nar/gkt402

**Published:** 2013-05-15

**Authors:** Tanit Guitart, Daria Picchioni, David Piñeyro, Lluís Ribas de Pouplana

**Affiliations:** ^1^Institute for Research in Biomedicine (IRB Barcelona), Gene Translation Laboratory, c/Baldiri Reixac 10, Barcelona, 08028, Catalonia, Spain and ^2^Catalan Institution for Research and Advanced Studies (ICREA), Pg. Lluís Companys 23, Barcelona, 08010, Catalonia, Spain

## Abstract

The translation of genes encoded in the mitochondrial genome requires specific machinery that functions in the organelle. Among the many mutations linked to human disease that affect mitochondrial translation, several are localized to nuclear genes coding for mitochondrial aminoacyl-transfer RNA synthetases. The molecular significance of these mutations is poorly understood, but it is expected to be similar to that of the mutations affecting mitochondrial transfer RNAs. To better understand the molecular features of diseases caused by these mutations, and to improve their diagnosis and therapeutics, we have constructed a *Drosophila melanogaster* model disrupting the mitochondrial seryl-tRNA synthetase by RNA interference. At the molecular level, the knockdown generates a reduction in transfer RNA serylation, which correlates with the severity of the phenotype observed. The silencing compromises viability, longevity, motility and tissue development. At the cellular level, the knockdown alters mitochondrial morphology, biogenesis and function, and induces lactic acidosis and reactive oxygen species accumulation. We report that administration of antioxidant compounds has a palliative effect of some of these phenotypes. In conclusion, the fly model generated in this work reproduces typical characteristics of pathologies caused by mutations in the mitochondrial aminoacylation system, and can be useful to assess therapeutic approaches.

## INTRODUCTION

Aminoacyl-tRNA synthetases (aaRSs) constitute an ancient family of enzymes that catalyze the attachment of amino acids onto their cognate transfer RNAs (tRNAs). The enzymes carry out a two-step reaction that first condenses the amino acid with ATP to form the aminoacyl adenylate and then transfer the aminoacyl moiety to the tRNA 3′ end (1). The aminoacyl-tRNA is then delivered to the ribosome by elongation factors for the decoding of the messenger RNA (mRNA) according to genetic code rules. In animals, as in the vast majority of eukaryotes, protein synthesis occurs simultaneously in the cytoplasm and some organelles that possess their own genome. Human mitochondria have a circular double-stranded DNA genome (mtDNA) that codes for 13 polypeptides that are components of the respiratory chain and the oxidative phosphorylation (OXPHOS), responsible for supplying energy to the cell. Additionally, human mtDNA codes for two ribosomal RNAs and the 22 mitochondrial tRNAs (mt-tRNAs) required to decode all human mitochondrial mRNA codons. To aminoacylate these 22 tRNAs, a whole set of nuclear-encoded aaRS needs to be imported and function inside the organelle.

Defects in elements involved in mitochondrial protein synthesis are related to a heterogeneous number of mitochondrial diseases, which show diverse clinical symptoms including deafness, blindness, encephalopathy and myopathy. More than 50% of the known mtDNA mutations are concentrated in tRNA genes and associated to a wide variety of ailments (2). For example, mutations in mt-tRNA^Leu^ (UAA) gene cause mitochondrial encephalomyopathy, lactic acidosis and stroke-like episodes (MELAS) (3,4), in mt-tRNA^Lys^ produce myoclonic epilepsy with ragged red fibers (MERRF) (5) and in tRNA^Ser^ typically lead to deafness (6). A poor understanding of the pathophysiology of mitochondrial translation diseases, the wide variety of symptoms they cause and the technical difficulty of working with mutant mitochondria complicate the research on these disorders. For that reason, the construction of model systems is necessary to facilitate the characterization, diagnosis and development of therapeutic approaches.

Possibly due to their essential role, mutations in aaRS are only described in rare diseases with low prevalence (7). Some types of the inherited peripheral neuropathy Charcot-Marie Tooth (CMT) and distal spinal muscular atrophy type V have been related to mutations in the genes coding for the cytoplasmic glycyl-, tyrosyl-, alanyl- and lysyl-tRNA synthetases (8–11). The pathogenic mechanisms for these disorders are not clear. In some cases a loss of aminoacylation activity and, in others, a toxic gain of function, have been proposed as the reasons behind the pathogenicity of these mutations (12–14). More recently, pathogenic mutations in nuclear-encoded proteins of the mitochondrial translation apparatus have also been described [reviewed in (7)], including mutations in aspartyl-, arginyl-, tyrosyl-, seryl-, histidyl-, alanyl-, methionyl-, glutamyl- and phenylalanyl-tRNA synthetases (15–23).

Some animal models for neurological conditions caused by mutations in cytoplasmic aaRS have been generated. For example, mice and *Drosophila melanogaster* have been used as models to study the CMT symptoms caused by substitutions in the glycyl-tRNA synthetase gene (*GARS*) (24–26) and tyrosyl-tRNA synthetase gene (*YARS*) (27). Similarly, the sequence identity between some human and *Saccharomyces cerevisiae* mt-tRNAs (28,29) has allowed the generation of yeast strains with mutations in mt-tRNA that mimic some human neuropathies such as MELAS (30). Trans-mitochondrial cybrid cell lines (31) have also been used to study the biochemical and cellular consequences of point mutations and deletions of mtDNA, including those affecting tRNA genes (32). A few animal models for mitochondrial illnesses caused by mutations in nuclear-encoded components of mitochondrial gene translation exist, such as the *D. melanogaster technical knockout* (*tko*) that carries a point mutation in the *MRPS12* nuclear gene encoding mitochondrial ribosomal protein S12 (33). Finally, allotropic expression of functional tRNA derivatives in cybrid cells holding MERRF or MELAS mutations (34–37), as well as overexpression of mitochondrial aaRS (38,39), have been tested as therapeutic approaches. Blastocyst injection of ES cell cybrids has allowed the creation of heteroplasmic trans-mitochondrial mice bearing mutant mtDNA from donor cells (40–43).

**Figure 1.**
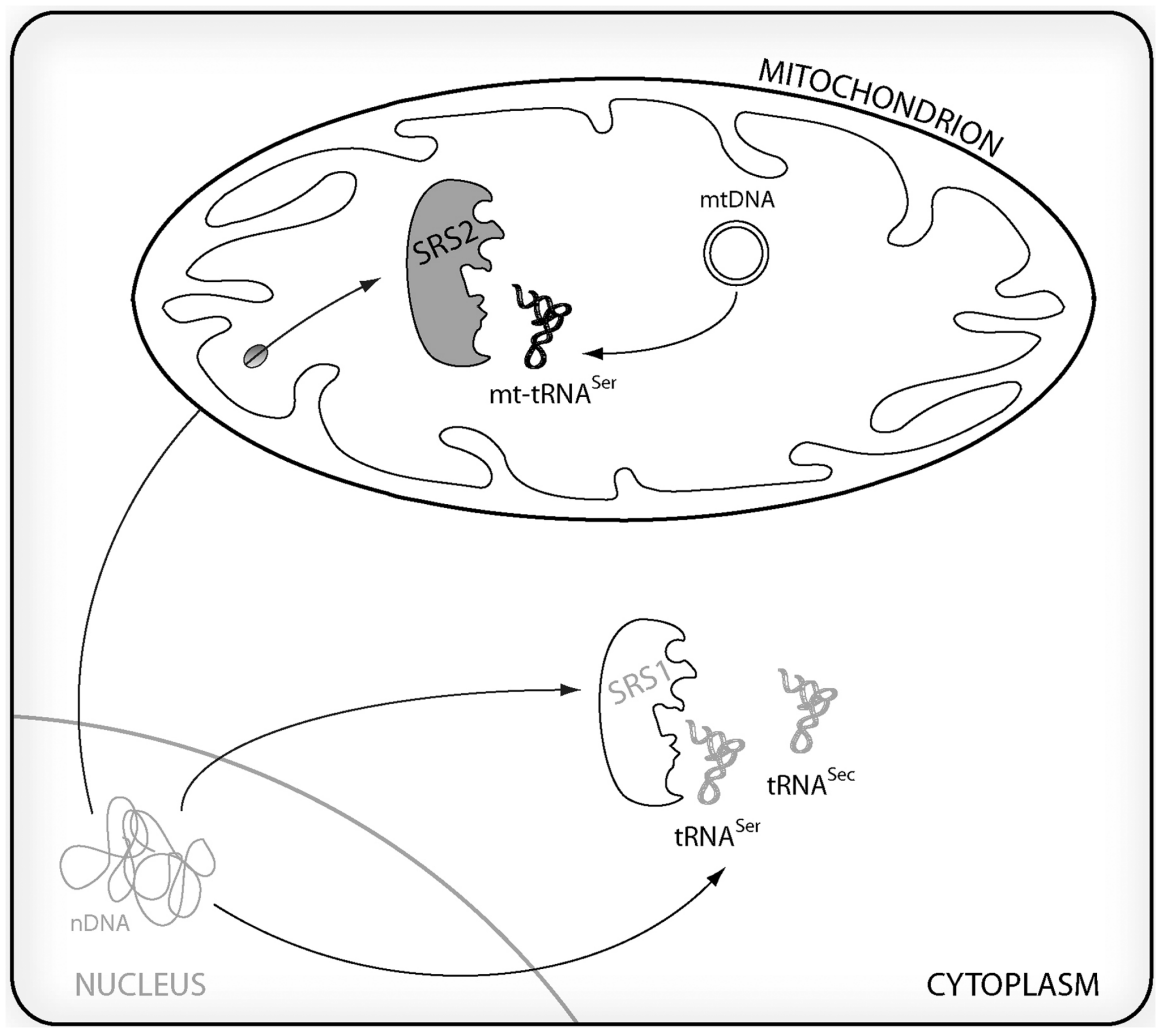
Animal serylation system. Nuclear genome contains two different seryl-tRNA synthetase (SRS) genes. SRS1 (in white) remains in the cytoplasm where it aminoacylates the nuclear-encoded tRNA^Ser^ and tRNA^Sec^ isoacceptors, while SRS2 (in gray) is led by means of a cleavable N-terminal targeting peptide to mitochondria, where it aminoacylates the two serine tRNAs encoded in the mitochondrial genome (mt-tRNA^Ser^).

The aim of the present work is the development of a *D. melanogaster* model to study the cellular and molecular effects of a deficient mt-tRNA aminoacylation activity. To achieve this goal, and avoid the drawbacks of manipulating mtDNA-encoded tRNAs, we have decided to limit the function of a nuclear-encoded mitochondrial aaRS, seryl-tRNA synthetase 2 (DmSRS2) (Figure 1), by means of an RNA interference (RNAi) approach. We have previously reported the identification of SRS2 in *D. melanogaster*, and its preliminary comparison with a paralogous protein present in insects (SLIMP) (44).

>

Here we describe the generation of a *D. melanogaster* model for human mitochondrial disease caused by mitochondrial aminoacylation restriction. The model has been first characterized at the molecular level, showing a decrease in DmSRS2 expression and function, with a reduction in mt-tRNA^Ser^ aminoacylation. Secondly, the effect of DmSRS2 general or tissue-restricted depletion has been analyzed. This insult compromises viability, longevity, motility and tissue development. At the cellular level, SRS2 silencing strongly affects mitochondrial morphology, biogenesis and function, and induces lactic acidosis and reactive oxygen species (ROS) accumulation. The later observation prompted us to investigate the effect of administering antioxidant molecules to the affected animals. We report that such a treatment has a palliative effect and reduces the severity of some of the phenotypes caused by the silencing of the enzyme.

## MATERIALS AND METHODS

### Fly maintenance and strains

Flies were maintained in 60% relative humidity in 12 h light/dark cycles at 18, 25 or 29°C depending on the experiment. Flies were fed with standard fly food except when testing the effect of antioxidant molecules in the phenotype. In these cases, the micronutrient supplement K-PAX (K-PAX Inc.) was added to fly’s food at a final concentration of 6.9 mg/ml. To determine the chromosomal location of the UAS-RNAi transgenes and for the proper manipulation of the transgenic flies, the balancer line *w*; *If/CyO*; *Ly/TM3-Sb* was used. UAS and GAL4 lines used in this study were as follows: *w*; *UAS-dicer-2*, *yw*; *actin5C-GAL4/TM6B*, *w*; *patched-GAL4*, *w*; *repo-GAL4/TM6B*, *yw*; *Mef2-GAL4*, and the *yw*; *nubbin-GAL4*; *UAS-dicer-2/CyO-TM6B* that ensures co-segregation of nubbin-GAL4 with UAS-*dcr2*.

### Generation of transgenic UAS-RNAi strains

A 543 bp fragment from the DmSRS2 cDNA was subcloned into pWIZ vector in an inverted repeat manner (45). Transgenic fly lines were obtained by microinjection of the construction into *w^1118^* embryos using standard procedures (46). One homozygous strain was obtained carrying the UAS-RNAi_DmSRS2_ transgene in chromosome II, which on crossing gave rise to the w; RNAi_DmSRS2_ strain 1-*dcr2* homozygous strain, that expressed both the RNAi_DmSRS2_ and dicer-2 protein. In addition, an independent strain carrying a different UAS-RNAi_DmSRS2_ transgene in chromosome II (RNAi_DmSRS2_ strain 23003) and a line used to silence the respiratory chain subunit ND75 from complex I (RNAi_ND75_), were purchased from the VDRC stock centre (ID 23003 and 100733, respectively) (47). Induction of RNAi transgene expression was based on the UAS-GAL4 system (48).

### Viability and life span determinations

To measure adult viability, crosses with the heterozygous actin5C-GAL4 driver were maintained at 25 and 29°C and progeny was counted to *n* > 150. The dsRNA was expected to be expressed in 50% of the progeny, while the remaining 50% should not produce it and was used as internal negative control. Adults with active RNAi_DmSRS2_ were counted and represented relative to the maximum expected viability, set as 100%. For life span experiments, crosses with repo-GAL4 driver were kept at 29°C and with Mef2-GAL4 driver at 18°C until adulthood to allow viability. For each experiment, ≥100 adults were collected, transferred to fresh food vials every two days, maintained at 29°C and counted daily. Survival curves were constructed and compared using the Log-rank (Mantel-Cox) method.

### Climbing assays

8–14 control flies (Mef2-GAL4), Mef2-GAL4; RNAi_DmSRS2_ strain 1-*dcr2* or RNAi_DmSRS2_ strain 23003 flies, hatched at 18°C, were collected in food vials at 29°C for 24 h. Climbing assays were performed as described (49), setting horizontal marks at 20, 50 and 100 mm height and giving flies 60 s to climb the vial.

### Quantitative real-time polymerase chain reaction

Total RNA was extracted from third instar larvae with TRIzol (Invitrogen), digested with DNase I and cleaned with the RNeasy MinElute Cleanup kit (Qiagen). One microgram of total RNA was retrotranscribed into cDNA using oligo(dT) primers to perform quantitative real-time polymerase chain reactions (PCRs) by means of *Power* SYBR Green and a StepOnePlus Real-time PCR System (Applied Biosystems). cDNA templates were amplified with a pair of primers designed with the Primer Express® software (Applied Biosystems) to detect the DmSRS2 cDNA (5′CCGTTCTGCGACCATTCAT3′ and 5′CAGCTTCGTCTCCGGTATCC3′) and another to detect the Rp49 cDNA, used as endogenous control (5′TGCCCACCGGATTCAAGA3′ and 5′AAACGCGGTTCTGCATGAG3′). Standard curves were calculated for both primer pairs to ensure a high efficiency level. Twenty microliters of reactions were prepared following the manufacturer’s instructions, using ROX as reference dye and the following conditions: 50°C for 2 min; 95°C for 10 min; 40 cycles (95°C for 15 s; 60°C for 1 min). Fold expression changes were calculated using the 2^−ΔΔ^^CT^ method, where ΔΔC_T_ is the RNAi ΔC_T_ [C_T_ average for DmSRS2 − C_T_ average for the reference gene (Rp49)] − the *w^1118^* control ΔC_T_ [C_T_ average for DmSRS2 − C_T_ average for the reference gene (Rp49)]. The value obtained for control larvae is represented as 1 and the other values are represented relative to it.

### Analysis of *in vivo* mt-tRNA^Ser^ aminoacylation

Total RNA was extracted with TRIzol (Invitrogen) from third instar larvae with inactive or active RNAi_DmSRS2_ and 30 μg of total RNA were electrophoresed on high-resolution acid gels, as described in (50) and transferred to a Hybond XL (GE Healthcare) membrane by vacuum gel drying transfer (51). Aminoacylated mt-tRNAs were analyzed by northern blot using the following radiolabeled probes: 5′TGGTCATTAGAAGTAAGTGCTAATTTAC3′ for mt-tRNA^Lys^ (CUU), used as a control, 5′TGGAGAAATATAAATGGAATTTAACC3′ for mt-tRNA^Ser^ (GCU) and 5′TGGAAGTTAATAGAAAATTAAATTCTATCTTATG3′ for mt-tRNA^Ser^ (UGA). Signals were digitalized using a PhosphorImager™ from a gel exposed storage phosphor screen and were quantified using the ImageQuant™ TL software (GE Healthcare).

### Wing preparation and microscopy image analyses

Twelve or more adults were kept at room temperature in 75% ethanol, 25% glycerol for >24 h and wings were excised in cold phosphate buffered saline (PBS) and mounted in Fauré’s medium. Images were taken in a Nikon E600 microscope with an Olympus DP72 camera, and L3-L4 areas were measured from males and females separately with the ImageJ software (52). Images from whole flies were taken at 30× with a MZ 16F Leica stereomicroscope equipped with a DFC 300FX camera.

### Electron microscopy

Fat bodies from 8 to 10 third instar larvae were dissected in Schneider’s medium and fixed in 2% glutaraldehyde in 0.1 M cacodylate buffer pH 7.2. Postfixation was done with 2% OsO_4_ and 1.6% K_3_Fe(CN)_6_ in cacodylate buffer. Sections were contrasted with uranyl acetate and visualized in a Jeol JEM 1010 electron microscope with a high-resolution digital camera. For mitochondrial surface determination, >70 mitochondria were measured, and for mitochondrial density calculation, 35 images at 20 000× were analyzed for each sample. The surface occupied by glycogen was calculated taking >15 images at 20 000× for each sample, and it was represented as the area covered by glycogen in μm^2^ in 100 μm^2^ of total area (subtracting the mitochondrial and lipid droplet surface). Image analyses were performed with the ImageJ software (52).

### mtDNA copy number determination

mtDNA was quantified as described (44).

### Lactate, glycogen and pH determination

Concentration of lactate in larval tissues was measured as described (53) with a Cobas Mira Plus analyzer (Roche). Concentration of glycogen in larval tissues was measured adapting the protocol described (54) to a Safire 2 fluorometer (Tecan Group Ltd.). For both experiments, 20 larvae were used for each determination, and results were normalized by total protein, measured using BCA Protein Assay Kit (Pierce). pH of larval homogenates was measured at 4°C with a pH meter GLP21 (Crison).

### Oxygen consumption measurements

Oxygen consumption was measured with an Oxygraph-2 k (Oroboros) as described (44). Complex I respiratory control ratios (RCR) were calculated using the oxygen consumption in the presence and absence of ADP (GM_D_ and GM). The oxygen flux values in different respiratory states were normalized dividing them by the mitochondrial rate calculated from the relative mtDNA quantification (55) considering the rate for control larvae as 1 and representing the other values relative to it.

### ROS assays

Wing imaginal discs were incubated with dihydroethidium (Invitrogen), rinsed twice in Schneider’s medium, fixed with 4% *P*-paraformaldehyde and washed with PBS (56). Superoxide anion accumulation was visualized by confocal imaging.

### Statistical analyses

All the statistical analysis were performed using the software GraphPad Prism version 5.00, except the two-way analysis of variance (ANOVA) test that was performed with the SPSS 15.0 software.

## RESULTS

### DmSRS2 knockdown causes an aminoacylation deficiency

**Figure 2.**
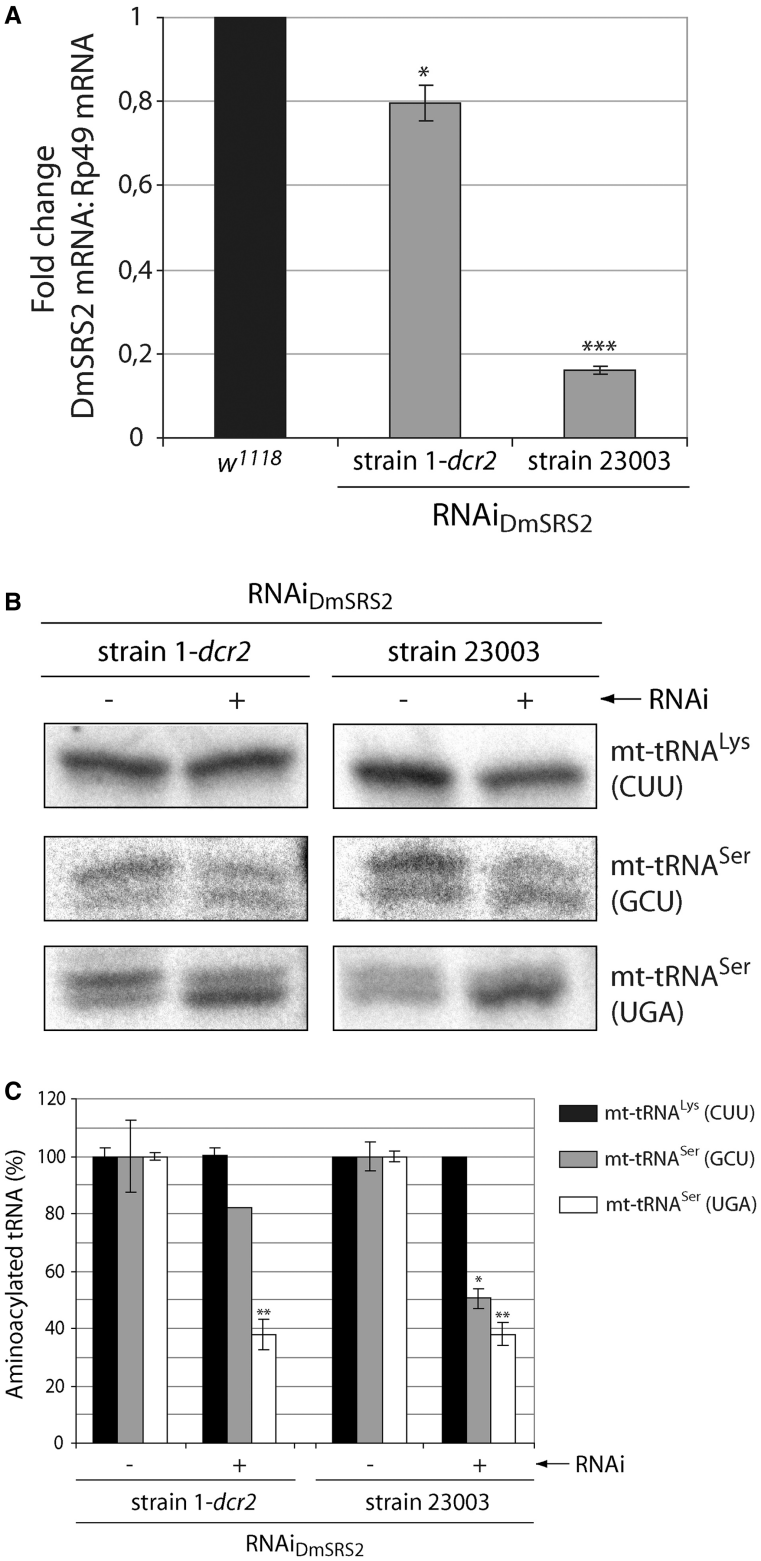
DmSRS2 knockdown strength. (**A**) DmSRS2 mRNA levels were quantified by real-time PCR in control larvae (*w^1118^*) and RNAi_DmSRS2_ larvae from strains 1-*dcr2* and 23003 crossed with actin5C-GAL4 driver at 29 and 25°C, respectively. DmSRS2 mRNA levels were normalized using Rp49 mRNA as reference. Graph gives average with SEM from three independent experiments, and statistical significance is calculated by Student’s *t*-test (*P < 0.05; ***P < 0.001). The mRNA level in control larvae is established as 1 and the other values are relative to this. (**B**) Aminoacylated and deacylated mitochondrial tRNAs (mt-tRNAs) were detected by northern blot from larvae with inactive (−) and active (+) RNAi for DmSRS2, coming from strain 1-*dcr2* and 23003 crossed with actin5C-GAL4 driver at 29 and 25°C, respectively. Thirty micrograms of total RNA were loaded into high-resolution acid gels, and probes were designed to specifically detect the two mitochondrial tRNA^Ser^ isoacceptors (GCU and UGA) and the mitochondrial tRNA^Lys^ (CUU) as control. (**C**) The graph shows, for each lane from panel *B*, the relative abundance of aminoacylated mt-tRNA^Lys^ (CUU) (black), mt-tRNA^Ser^ (GCU) (gray) and mt-tRNA^Ser^ (UGA) (white), setting the levels of aminoacylated mt-tRNAs in larvae with inactive (−) RNAi as 100%. The mean from two independent experiments with SEM are represented and subjected to Student’s *t*-test (*P < 0.05; **P < 0.01).

To partially reduce the levels of the *D. melanogaster* SRS2 in an inducible and regulated manner, we used RNAi expression under the control of the UAS-GAL4 system. We used two fly strains holding two RNAi transgenes designed to specifically target two different regions of the DmSRS2 mRNA (RNAi_DmSRS2_ strain 1-*dcr2* and RNAi_DmSRS2_ strain 23003). When the RNAi_DmSRS2_ strains were crossed with the actin5C-GAL4 driver, the expression of the RNAi transgenes was constitutively and ubiquitously induced in the offspring. Because GAL4 activity is temperature dependent, crosses with actin5C-GAL4 were maintained at the highest temperature that allowed viability of the progeny, to ensure high efficiency of the RNAi silencing. Thus, the cross with RNAi_DmSRS2_ strain 1-*dcr2* was maintained at 29°C, while the one with RNAi_DmSRS2_ strain 23003 was kept at 25°C. Both RNAi transgenes produced a reduction in DmSRS2 mRNA levels in larvae (Figure 2A), with different efficiencies depending on the strain used: RNAi_DmSRS2_ strain 1-*dcr2* showed a mild effect with limited reduction to 0.796 ± 0.040, and RNAi_DmSRS2_ strain 23003 displayed a strong effect with a marked decrease to 0.163 ± 0.001, while the mRNA levels of the cytosolic DmSRS (DmSRS1) and the DmSRS2 paralogous SLIMP did not change significantly (Supplementary Figure S1). The availability of two strains with different efficiencies to silence DmSRS2 allowed us to investigate the range of phenotypes resulting from different degrees of silencing. This is reminiscent of the different severity levels and variety of symptoms that occur in most mitochondrial diseases.

>

To prove that the RNAi_DmSRS2_ was decreasing the function of the enzyme, the relative levels of aminoacylated and deacylated mitochondrial tRNA^Ser^ (mt-tRNA^Ser^) from larvae were analyzed by northern blot (Figure 2B), and the levels of *in vivo* aminoacylation under general RNAi induction were quantified and compared with RNAi inactive larvae (Figure 2C).

In Figure 2B, intensity of the upper bands (corresponding to aminoacylated tRNAs) for the two mt-tRNA^Ser^ isoacceptors (GCU and UGA) was reduced when RNAi was functional (+), while mt-tRNA^Lys^ (CUU) was maintained completely aminoacylated, showing a single band (Supplementary Figure S2). RNAi_DmSRS2_ (+) larvae from strain 1-*dcr2* showed a moderate decrease (Figure 2C) in mt-tRNA^Ser^ (GCU) aminoacylation level to 82.10 ± 0.01%, while larvae from strain 23003 showed a strong reduction to 50.44 ± 3.31%. Levels of mt-tRNA^Ser^ (UGA) aminoacylation were similar in both strains when RNAi was functional: 37.88 ± 5.3% (strain 1-*dcr2*) and 38.06 ± 4.14% (strain 23003). Indeed, we observed a reduction of the mitochondrial encoded proteins ND1 (MT-ND1) and COX2 (MT-CO2) in knockdown larvae from RNAi_DmSRS2_ strain 1-*dcr2* at 29°C (5.2% and 43.6%, respectively) and strain 23003 at 25°C (48.7 and 51.3%, respectively) compared with the control *w^1118^* (Supplementary Figure S3). These results confirmed that DmSRS2 was the *D. melanogaster* mitochondrial seryl-tRNA synthetase, as its silencing specifically diminished the amount of seryl-mt-tRNA^Ser^ and mitochondrially encoded proteins. Therefore, the RNAi of DmSRS2 was confirmed to be a useful approach to limit the mitochondrial translation capacity in a controlled manner.

### DmSRS2 silencing influences viability and tissue development

**Figure 3.**
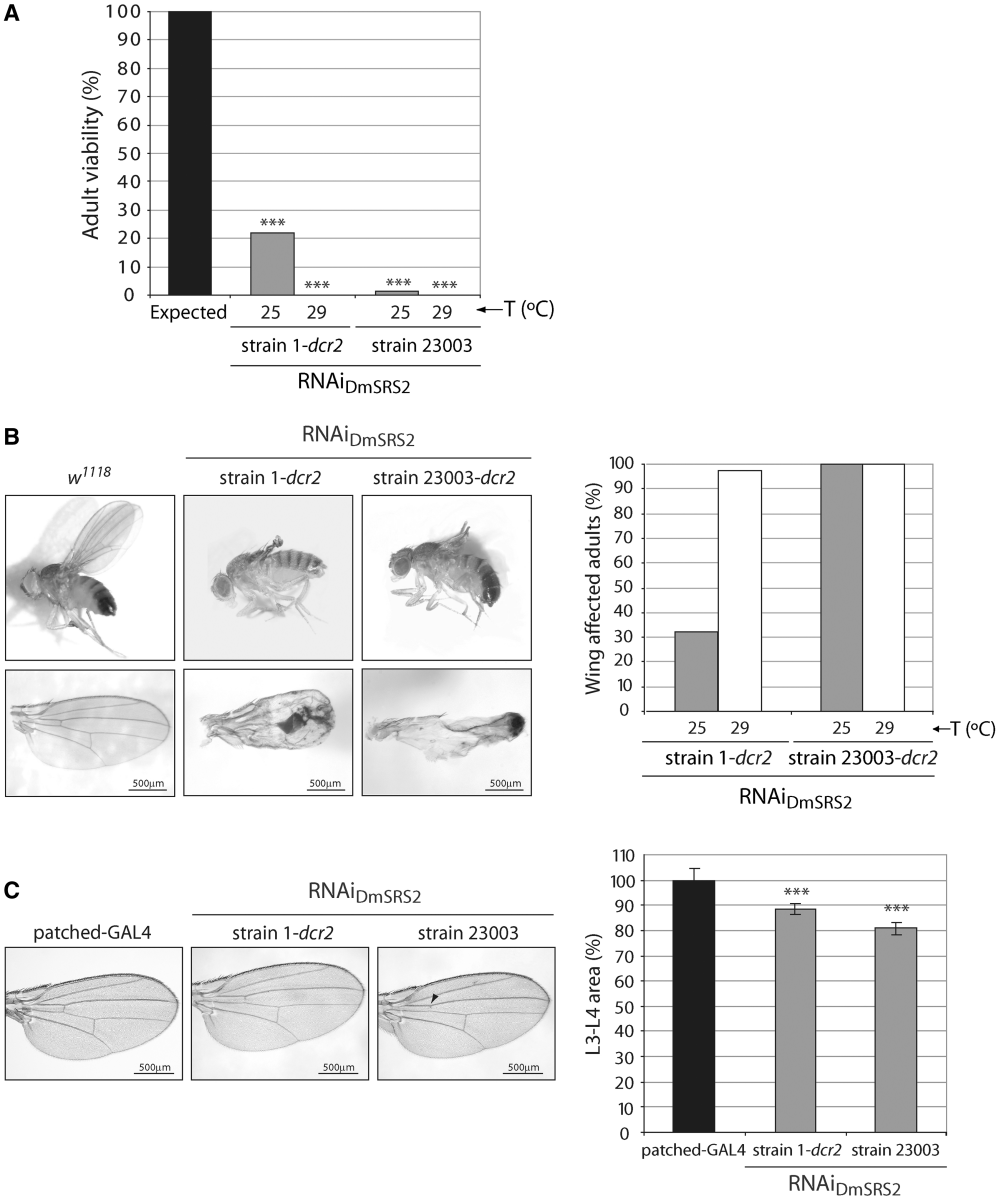
*In vivo* effect of RNAi of DmSRS2. (**A**) Adult viability reduction caused by the constitutive and ubiquitous silencing of DmSRS2 at 25 and 29°C using the two RNAi_DmSRS2_ strains. Results are represented setting the maximum expected viability of RNAi active flies at 100%. Statistical significance is determined by Chi-square test (***P < 0.001). (**B**), DmSRS2 knockdown restricted to wing. The left panel shows images of adults from the crosses between nubbin-GAL4; UAS-*dcr2* driver and RNAi_DmSRS2_ strain 1 and strain 23003, which suffer severe wing damage, and control flies (*w^1118^*). Scale bars correspond to 500 μm. Graph on the right represents the proportion of adults that exhibit wing defects when crosses are kept at 25 and 29°C. (**C**), Patched-GAL4 driver is crossed at 29°C with RNAi strains to restrict the DmSRS2 depletion in the region flanked by longitudinal veins L3 and L4. The images on the left show wings with a partial or total loss of the anterior cross vein (marked with an arrowhead) and a reduction in the L3-L4 area, compared with the parental line (patched-GAL4). Scale bars correspond to 500 μm. Graph on the right shows the averages with SEM of all the L3-L4 area measurements in percentage, compared with the parental line and statistics are performed by two-way ANOVA test (***P < 0.001).

To study the phenotype caused by DmSRS2 depletion, we crossed the actin5C-GAL4 driver with the two transgenic RNAi_DmSRS2_ strains. At 25°C, 21.8% of strain 1-*dcr2* pupae, and 1.4% of strain 23003 pupae, hatched successfully. At 29°C, temperature at which GAL4 activity is maximum (57), no adult animals could be observed to hatch from pupae (Figure 3A). As expected for an aaRS, silencing of DmSRS2 impedes viability.

>

We used tissue-restricted RNAi induction to investigate effects caused by DmSRS2 silencing without compromising the viability of the organisms (Figure 3B and C). To express the RNAi in the wing imaginal disc region, which afterward develops into the adult wing blade and hinge, we crossed the RNAi_DmSRS2_ strains with nubbin-GAL4; UAS-*dcr2* driver (Figure 3B). As shown in the right panel, a 32.3% (25°C) and a 97.7% (29°C) of RNAi_DmSRS2_ strain 1-*dcr2* flies showed tissue damage in wings, while all the RNAi_DmSRS2_ strain 23003-*dcr2* flies presented wing defects both at 25 and 29°C. Although the wings conserved their general structure, the blade was unable to unfold and develop completely (left panel).

To analyze the cellular effects of DmSRS2 silencing in the wing, we crossed the RNAi transgenic lines with patched-GAL4 driver at 29°C, which allows for a better definition of the RNAi expressing region (Figure 3C). The gene *patched* is expressed in the anteroposterior border of the wing disc, which gives rise to the wing area limited by longitudinal veins 3 (L3) and 4 (L4) (58). A partial or total loss of the anterior cross vein (acv) was observed in 4 and 15% of the wings from flies emerging from the crosses with strain 1-*dcr2* and strain 23003, respectively. Moreover, in these animals, the L3-L4 wing area underwent a significant narrowing of 88.58 ± 2.13% (strain 1-*dcr2*) and 80.83 ± 2.54% (strain 23003), compared with the parental strain (patched-GAL4). The reduction in L3-L4 area was not due to a decrease in cell size, but in cell number, although there was no evidence of apoptosis when wing imaginal discs were subjected to Caspase-3 immunofluorescence (data not shown). Because the L2-L3 area, contiguous to L3-L4, did not show a significant decrease (data not shown), the effect observed in the L3-L4 region was considered cell autonomous.

### Life span and motility are reduced by DmSRS2 knockdown

**Figure 4.**
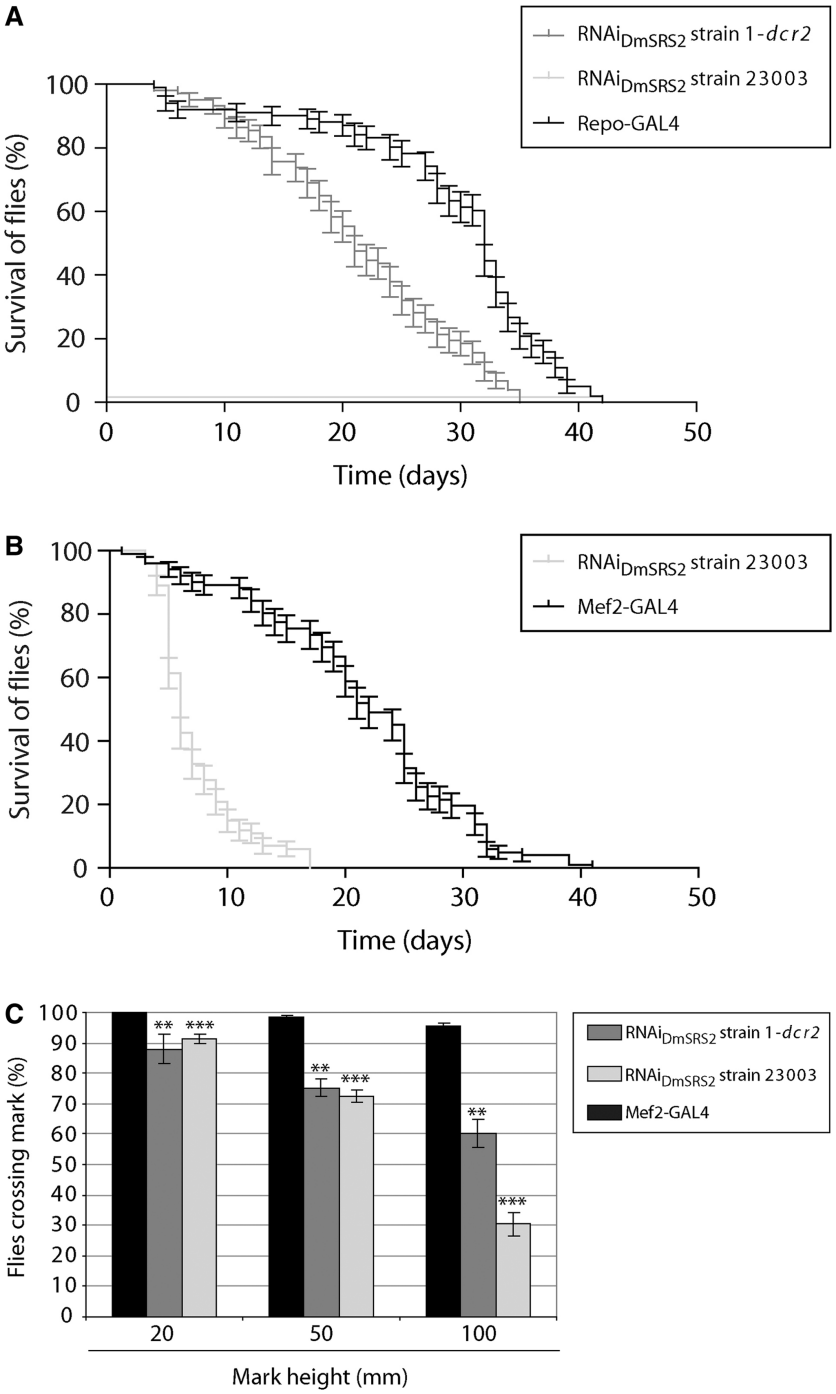
DmSRS2 knockdown repercussion in longevity and locomotion ability. (**A**) Life span at 29°C of flies with RNAi_DmSRS2_ restricted to glial cells, compared with the parental line (repo-GAL4). (**B**) Muscle-restricted DmSRS2 silencing using RNAi_DmSRS2_ 23003 compared with parental line (Mef2-GAL4) shortens life span (Log-rank test *P* < 0.0001) and half-life from 22 to 6 days. Individuals where maintained at 18°C until adulthood when they were switched to 29°C. (**C**) Climbing ability is compromised in adults emerging from crosses between Mef2-GAL4 and RNAi_DmSRS2_ strain 1-*dcr2* or RNAi_DmSRS2_ strain 23003, according to that for the parental flies (Mef2-GAL4). Values from at least four assays for each genotype were averaged, represented with SEM and compared by Student’s *t*-test (**P < 0.01, ***P < 0.001).

With the purpose of disrupting *Drosophila* mitochondrial translation in tissues typically affected in patients with mitochondrial pathologies, DmSRS2 silencing was restricted to neural and muscle cell types using repo-GAL4 and Mef2-GAL4 drivers, respectively (Figure 4). In Figure 4A, the survival curve for RNAi_DmSRS2_ strain 1-*dcr2* flies with a repo-GAL4 driver at 29°C was significantly different to the parental line repo-GAL4, with a reduction in half-life from 32 to 21 days. Furthermore, repo-GAL4; RNAi_DmSRS2_ strain 23003 individuals were unable to hatch from pupal stage, at any tested temperature (18, 25 and 29°C).

>

To obtain adult animals from the Mef2-GAL4 × RNAi_DmSRS2_ strain 1-*dcr2* and the Mef2-GAL4 × RNAi_DmSRS2_ strain 23003 crosses, progeny was maintained at 18°C and adult life span was compared with control line Mef2-GAL4. Muscle-restricted DmSRS2 silencing led to a shortening in life span with half-life of 6 days, compared with 22 days of the control (Figure 4B). The RNAi_DmSRS2_ induction in muscle dramatically affected adult locomotion capacity when compared with parental strain Mef2-GAL4. In standard climbing assays, flies from the parental line showed 100% at 20 mm, 98.38 ± 0.70% at 50 mm and 95.59 ± 0.98% at 100 mm, RNAi_DmSRS2_ strain 1-*dcr2* flies showed a decrease to 87.90 ± 4.79% at 20 mm, 75.22 ± 2.88% at 50 mm, 60.27 ± 4.70% at 100 mm and RNAi_DmSRS2_ strain 23003 adults showed a reduction to 91.41 ± 1.62% at 20 mm, 72.31 ± 2.03% at 50 mm, 30.42 ± 3.91% at 100 mm (Figure 4C).

### DmSRS2 depletion alters mitochondrial morphology and biogenesis

**Figure 5.**
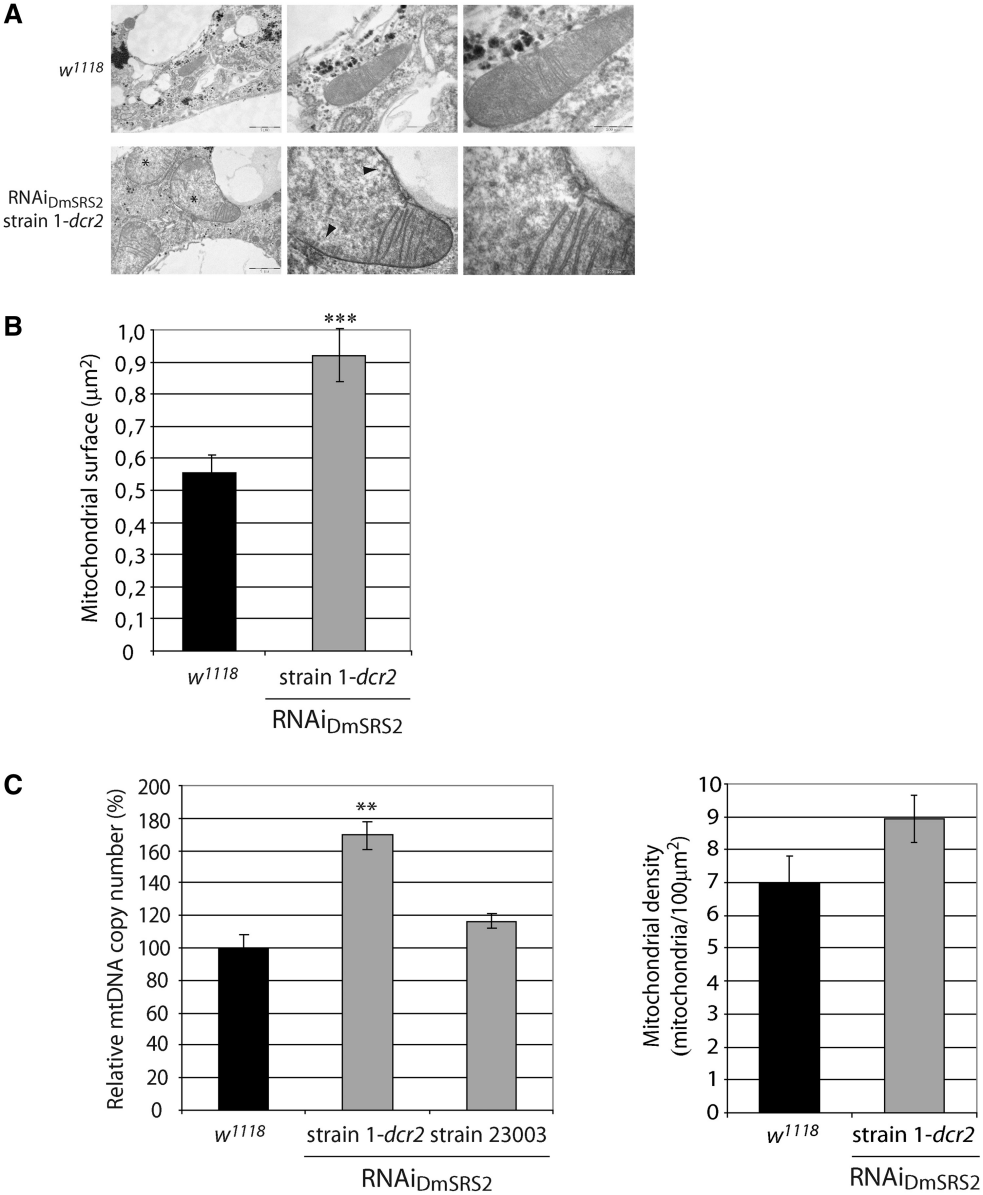
RNAi_DmSRS2_ effect in mitochondrial morphology and biogenesis. (**A**) Micrographs of fat bodies from control larvae (*w^1118^*) and larvae under RNAi_DmSRS2_ constitutive and ubiquitous activation at 29°C. Affected mitochondria show electron pale and swollen matrices (marked with *) and a loss of mitochondrial cristae (marked with arrowheads). Scale bars correspond to 1 μm, 500 nm and 200 nm, from left to right. (**B**) TEM images were used to measure mitochondrial surface in *w^1118^* and RNAi_DmSRS2_ strain 1-*dcr2* fat bodies. Columns represent the mitochondrial surface mean with SEM, and Student *t*-test is performed to determine statistical significance (***P < 0.001). (**C**) The left graph shows the relative mtDNA copy number determination in control larvae (*w^1118^*) and larvae from the cross between strains RNAi_DmSRS2_ 1-*dcr2* or 23003 and actin5C-GAL4 at 29 and 25°C, respectively. Columns represent the average of more than three replicates with SEM, where relative mtDNA copy number in control larvae is set as 100% and the percentages for the affected larvae are expressed relative to it (Student’s *t*-test **P < 0.01). The graph in the right shows the mitochondrial density calculated from TEM images in *w^1118^* and RNAi_DmSRS2_ strain 1-*dcr2* larval fat bodies.

After the characterization of tissue and cellular defects caused by DmSRS2 silencing, we aimed to determine the effects at the subcellular level. Fat bodies from control larvae, or from the cross between actin5C-GAL4 and RNAi_DmSRS2_ strain 1-*dcr2*_,_ at 29°C, were visualized by transmission electron microscopy (TEM). SRS2 silencing notably affected mitochondrial ultrastructure (Figure 5A) compared with control mitochondria. Mitochondria under DmSRS2 depletion were characterized by swollen matrices with low electron density, an evident reduction of cristae and a significant enlargement (Figure 5B), with an average mitochondrial surface of 0.920 ± 0.08 μm^2^, which represents a 66% increase relative to *wt* mitochondrial area (0.555 ± 0.05 μm^2^).

>

To evaluate the effect on mitochondrial biogenesis of RNAi_DmSRS_ expression, density of mitochondria was estimated by the quantification of relative mtDNA copy numbers. Larvae under DmSRS2 depletion showed an increase in mtDNA copy number (Figure 5C left panel). We observed a rise to 169.30 ± 8.61% when using RNAi_DmSRS2_ strain 1-*dcr2* as parental line (at 29°C) and to 116.60 ± 4.39% when using strain 23003 (at 25°C). These results are in agreement with an increase in mitochondrial number observed in the electron micrographs, in which RNAi_DmSRS2_ affected adipocytes possessed 8.94 ± 0.73 mitochondria/100 μm^2^ of cell surface, compared with 7.01 ± 0.79 mitochondria/100 μm^2^ of cell surface in wild-type cells (Figure 5C right panel).

### Mitochondrial translation deficiency leads to lactic acidosis and reduction of glycogen

**Figure 6.**
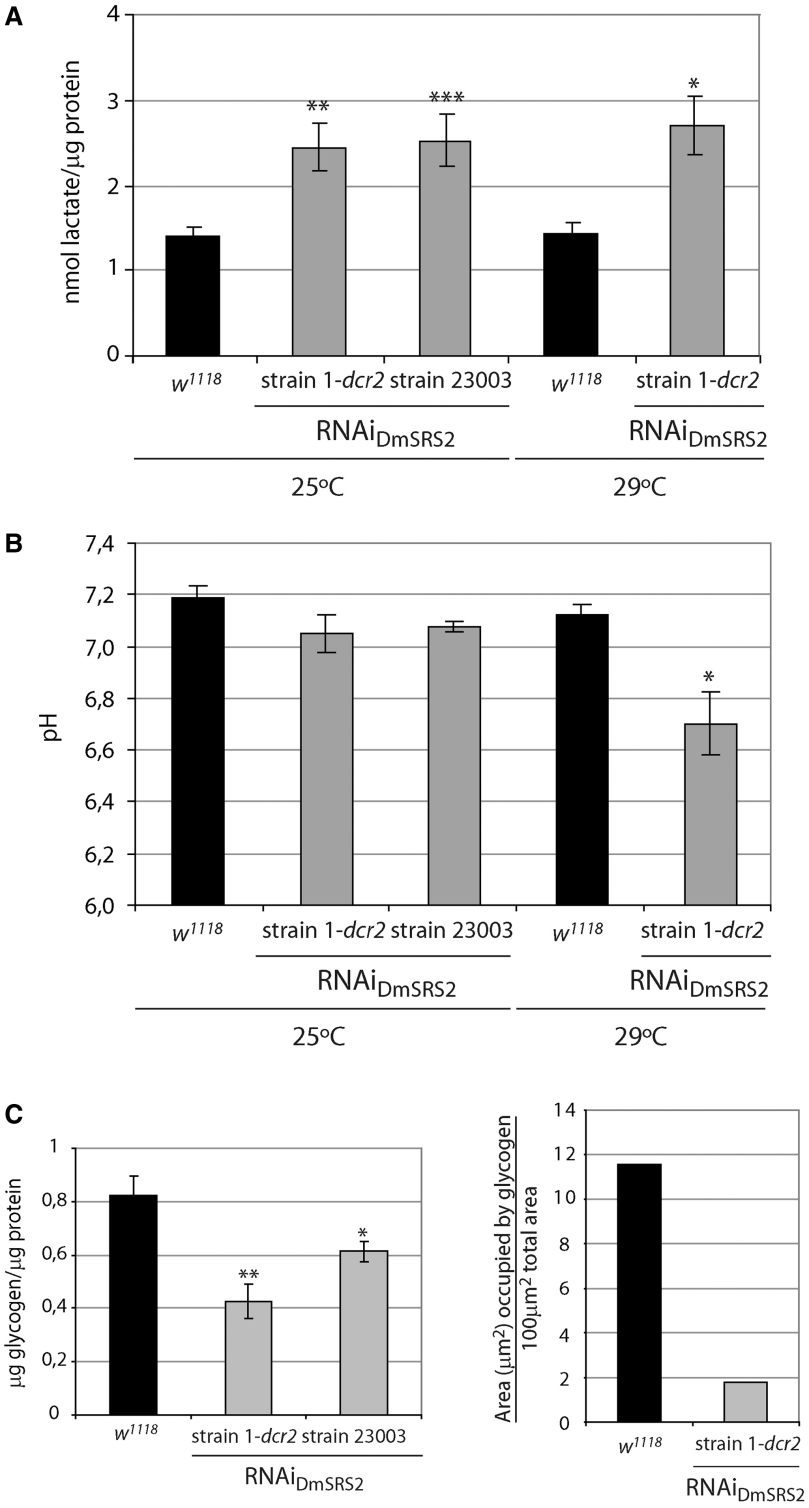
Metabolic consequences of DmSRS2 reduction. (**A**) Lactate concentration in larval tissue, which expresses the RNAi_DmSRS2_ with the actin5C expression pattern, compared with control (*w^1118^*) larval tissue, at 25 and 29°C. Values from seven independent determinations were averaged, represented in the graph with SEM and evaluated by Student’s *t*-test (*P < 0.05, **P < 0.01, ***P < 0.001). (**B**) Tissular pH from control and DmSRS2 ubiquitously and constitutively depleted larvae at different temperatures. Columns represent the mean of more than three replicates with SEM, where significance is determined by Student’s *t*-test (*P < 0.05). (**C**) On the left graph, glycogen concentration in larval tissue subjected to RNA_DmSRS2_ general expression, compared with control (*w^1118^*) larval tissue, at 25°C. Columns show the average with SEM from at least four measurements and analyzed by Student’s *t*-test (*P < 0.1, **P < 0.01). On the right graph, electron micrographs from *w^1118^* and RNAi_DmSRS2_ strain 1-*dcr2* larval fat bodies were used to determine the area occupied by glycogen.

One of the first signs of mitochondrial pathology in patients is the appearance of lactic acidosis, a physiological state caused by mitochondrial metabolism deficiency. Low levels of ATP synthesis result in the accumulation of cytosolic pyruvate, which is converted to lactate to satisfy energy demands (59). Simultaneously, inefficient mitochondrial respiration leads to the acidification of blood and tissues. Lactate concentration in samples from control flies had lactate values of 1.41 ± 0.11 nmol/μg protein, and 1.44 ± 0.11 nmol/μg protein, at 25 and 29°C, respectively (Figure 6A). In larval tissues under RNAi_DmSRS2_ activation, this concentration rose to 2.44 ± 0.27 nmol/μg protein and 2.7 ± 0.34 nmol/μg protein at 25 and 29°C, for strain 1-*dcr2*. Similarly, strain 23003 had lactate values of 2.53 ± 0.3 nmol/μg protein at 25°C. In agreement with these findings, the pH of DmSRS2-silenced larvae decreased from 7.19 ± 0.04 in *w^1118^*, to 7.05 ± 0.07 in RNAi_DmSRS2_ strain 1-*dcr2*, and to 7.07 ± 0.02 in strain 23003, at 25°C. Moreover, larvae maintained at 29°C showed a decrease in pH from 7.12 ± 0.04 to 6.70 ± 0.12 in RNAi_DmSRS2_ strain 1-*dcr2* at 29°C (Figure 6B).

>

Larval glycogen levels for control flies were also measured, obtaining a concentration of 0.82 ± 0.07 µg/µg protein at 25°C. The larval glycogen concentration at 25°C from strain 1-*dcr2* and strain 23003 were 0.42 ± 0.06 µg/µg protein and 0.61 ± 0.04 µg/µg protein, respectively (Figure 6C, left panel). These results are in agreement with a reduction in the area occupied by glycogen observed in the electron micrographs (Figure 6C, right panel). Thus, the silencing of DmSRS2 leads to a statistically significant reduction in cellular glycogen.

Taken together, our results may indicate that an increase in lactate production and a reduction in stored glycogen are linked consequences of a decreased capacity for mitochondrial protein synthesis.

### RNAi_DmSRS2_ compromises mitochondrial respiration and triggers ROS build-up

**Figure 7.**
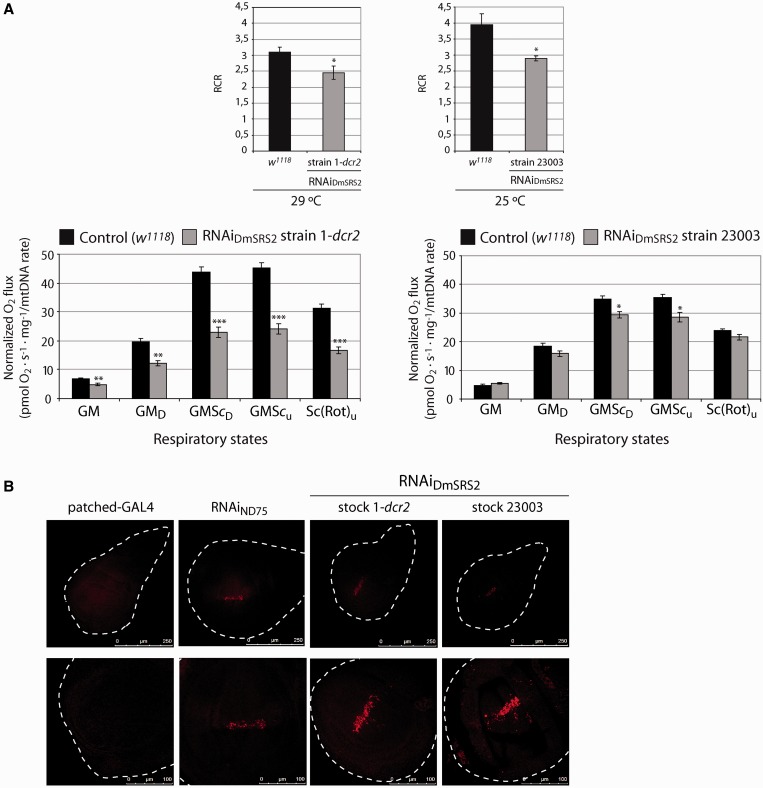
RNAi_DmSRS2_ effect in mitochondrial function. (**A**) Upper graphs represent the complex I RCRs (state 3 GM_D_/state 2 GM oxygen consumption) of mitochondria from larvae emerging from the crosses between actin5C-GAL4 and RNAi_DmSRS2_ strain 1-*dcr2* or strain 23003 at 29 and 25°C, respectively, compared with that from control (*w^1118^*) at the corresponding temperature. Average RCRs are calculated using oxygen consumption values. Lower graphs show the mitochondrial oxygen consumption profile from larvae under constitutive and ubiquitous induction of RNAi_DmSRS2_. Values normalized by mitochondrial density show a decrease in the mitochondrial respiration for RNAi_DmSRS2_ strain 1-*dcr2* and 23003. Graphs give the mean of oxygen flux determinations from more than three experiments with SEM (*P < 0.05, **P < 0.01, ***P < 0.001). (**B**) Confocal sections of wing imaginal discs from parental larvae or larvae emerging from patched-GAL4 crossed at 29°C with the two RNAi_DmSRS2_ strains and with an RNAi_ND75_ strain, used as positive control (56). Cells located in the zone where RNAi is restricted show an increase in fluorescence, which indicates the accumulation of superoxide anion. Upper images show a general view and lower images focus on the affected zones (scale bars correspond to 250 and 100 μm, respectively).

Because mitochondrial morphology, biogenesis and metabolism were altered in DmSRS2-silenced larvae, we decided to test if they were accompanied by a decrease in mitochondrial respiratory capacity. We monitored the mitochondrial oxygen consumption of larval tissues on addition of substrates and inhibitors of the respiratory chain and OXPHOS complexes (Figure 7A). Complex I RCR [oxygen consumption under ADP addition (GM_D_; state 3) divided by oxygen consumption limited by ADP (GM; state 2)] in larvae subjected to DmSRS2 general knockdown were lower than in control animals, indicating an uncoupling between the respiratory chain and OXPHOS (60). The RCR for RNAi_DmSRS2_ strain 1-*dcr2* mitochondria showed a decrease to 2.45 ± 0.20, compared with control (3.10 ± 0.17) at 29°C and for RNAi_DmSRS2_ strain 23003 a reduction to 2.89 ± 0.07, in comparison with 3.95 ± 0.34 in *w^1118^* at 25°C (Figure 7A, upper graphs). In larvae obtained from crosses between actin5C-GAL4 line and RNAi_DmSRS2_ strain 1-*dcr2* (at 29°C) or strain 23003 (at 25°C), oxygen consumption values (normalized by mitochondrial density) showed a notable decrease, and thus the respiratory capacity per mitochondria is lower in RNAi_DmSRS2_ affected tissues (Figure 7A, lower graphs). The increment in mitochondrial surface and number might be a compensatory response to the limited mitochondrial function caused by the DmSRS2 depletion.

>

To synthesize ATP by OXPHOS, the mitochondrial respiratory chain complexes transport electrons that are finally transferred to the molecular oxygen. When the respiratory chain and OXPHOS function incorrectly, ROS accumulate inside mitochondria and can induce oxidative stress. Taking into account that DmSRS2 is crucial for mitochondrial activity, we checked if the restricted expression of the RNAi_DmSRS2_ in the anteroposterior border of the wing imaginal disc (using patched-GAL4 driver) led to an increase in ROS. As shown in Figure 7B, wing imaginal discs exhibited a marked increase in superoxide anion restricted to cells under DmSRS2 interference at 29°C, comparable with that of cells affected by an RNAi against the ND75 complex I subunit of the respiratory chain, used as positive control.

### Antioxidant treatment palliates defects caused by RNAi_DmSRS2_

**Figure 8.**
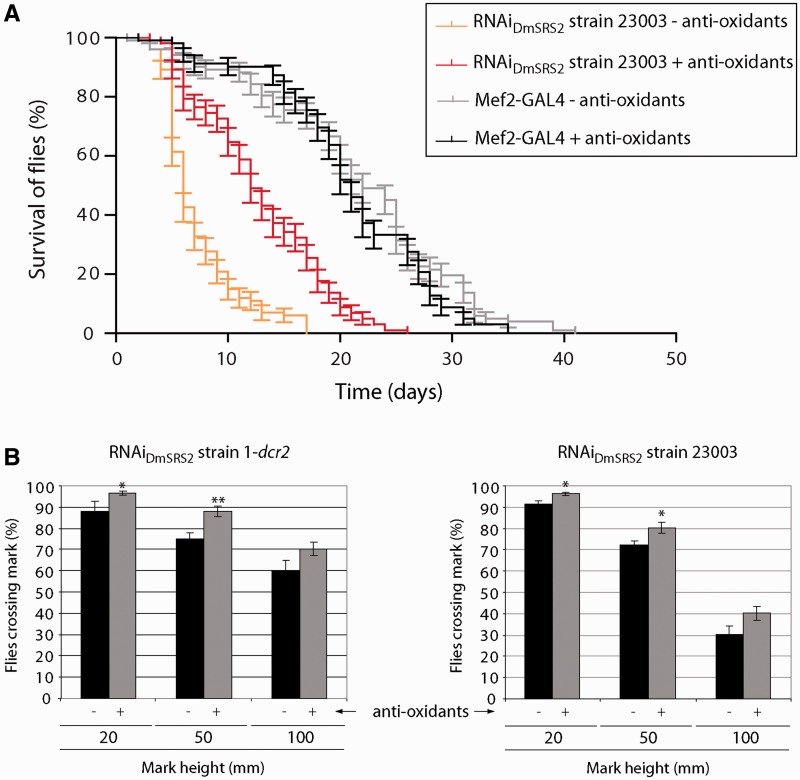
Palliative effect of antioxidant molecules. (**A**) Longevity of adults with muscle restricted RNAi_DmSRS2_, maintained at 29°C, increases significantly when antioxidant compounds are added to the diet (Log-rank test *P* < 0.001). Half-life rises from 6 to 12 days. (**B**) Adults that emerge from the crosses between Mef2-GAL4 and RNAi_DmSRS2_ strain 1-*dcr2* (left panel) or RNAi_DmSRS2_ strain 23003 (right panel) improve their climbing ability when they are treated with antioxidant molecules. Columns show the average with SEM for at least four assays performed for each condition and analyzed by Student’s *t*-test (*P < 0.05, **P < 0.01).

Considering that reduction in mitochondrial tRNA^Ser^ serylation disturbs organelle function and increases ROS production, we investigated whether supplementing flies’ diet with an antioxidant cocktail (K-PAX; K-PAX Inc.) may have a palliative effect on affected flies. Indeed, adult flies with muscle-specific RNAi_DmSRS2_ silencing at 29°C underwent a significant improvement in longevity (a half-life increase from 6 to 12 days) when treated with the antioxidant mix (Figure 8A). Similarly, supplementation with antioxidant molecules improved locomotion ability in both the RNAi_DmSRS2_ 1-*dcr2* and the RNAi_DmSRS2_ 23003 strains (Figure 8B). In the case of RNAi_DmSRS2_ 1-*dcr2* adults, 96.45 ± 0.92% were able to pass the 20 mm mark (untreated 87.90 ± 4.79%), 88.03 ± 2.29% passed the 50 mm mark (untreated 75.22 ± 2.88%) and 70.24 ± 3.12% passed the 100 mm mark (untreated 60.27 ± 4.70%) (left panel). Among RNAi_DmSRS2_ strain 23003 flies, 96.26 ± 0.91% were able to pass the 20 mm mark (untreated 91.41 ± 1.62%), 80.17 ± 2.45% passed the 50 mm mark (untreated 72.31 ± 2.03%) and 40.15 ± 3.15 % passed the 100 mm mark (untreated 30.42 ± 3.91%) (right panel).

>

## DISCUSSION

As predicted, silencing of SRS2 expression by means of RNAi in *D. melanogaster* led to a decrease in the aminoacylation levels of the two mitochondrial tRNA^Ser^. This fact confirmed the DmSRS2 canonical function as mitochondrial seryl-tRNA synthetase *in vivo* [previously proposed by bioinformatic approaches in (44)], and allowed us to generate an animal model for human mitochondrial disease caused by aminoacylation deficiencies.

The stringency of the system was modulated using two different RNAi transgenic strains, crossing them with driver lines with various promoters and maintaining the animals at different temperatures. In that way, RNAi_DmSRS2_ strain 23003 resulted more efficient in targeting DmSRS2 mRNA and reducing serine-mt-tRNA^Ser^ levels compared with RNAi_DmSRS2_ strain 1-*dcr2* (Figure 2). Accordingly, the constitutive or tissue-restricted silencing using strain 23003 produced strongest consequences in adult viability, longevity and tissue development (Figures 3 and 4).

This approach allowed us to induce DmSRS2 silencing, and produce flies with different levels of protein depletion leading to a range of phenotype severity, which permitted a better reproduction of the variability of symptoms displayed by patients with mitochondrial disorders. This is particularly true of those symptoms caused by mutations in mt-tRNAs that cause MELAS or MERRF.

These pathologies are characterized by lactate acidosis and mitochondrial respiratory chain dysfunction (16–18,20,22,23), traits also present in our *Drosophila* model (Figures 6A, B and 7A). The drop in the complex I RCR, caused by an increase in the oxygen consumption in state 2, indicated an uncoupling between the respiratory chain and the OXPHOS, possibly due to an anomalous permeabilization of the mitochondrial inner membrane to protons, which could positively feedback the respiratory chain independently of the F_1_-F_0_-ATPase activity (60). Moreover, oxygen consumption values in the different respiratory states suffered a clear reduction when they were normalized taking into account the mitochondrial content (55). These results suggest that affected tissues in our model suffer from a defective mitochondrial respiratory capacity that is masked by an increase in mitochondrial biogenesis, a compensatory response also observed in patients with MELAS/MERRF symptoms (61–63). Intracellular ROS have been proposed as modulators of mitochondrial proliferation (64). Similarly, DmSRS2 deficiency might induce an increase in mitochondrial biogenesis stimulated by the accumulation in ROS (Figure 7C).

The mitochondria of MELAS and MERRF patients present varying degrees of heteroplasmy, that is, the coexistence of different proportions of wild-type and mutant mtDNA populations in different tissues. Pathological symptoms appear when the mutant mtDNA copy number exceeds the level that guarantees the correct functioning of a tissue (threshold effect), leading to a complex variety of clinical manifestations with different levels of severity. This situation is again mimicked by our model, whose mitochondria suffer from a partial ablation of DmSRS2 activity. The affected organelles appear enlarged, with a decrease in the surface occupied by cristae, and electron pale matrices (Figure 5). Apart from morphological abnormalities in mitochondria, the RNAi_DmSRS2_ prompted an increase in mitochondrial density, observed in the micrographs and confirmed by relative mtDNA quantification.

Taking into account the DmSRS2 silencing generated oxidative stress, and with the aim to asses a therapeutic approach to palliate the defects in longevity and locomotion ability caused by the muscular deficit of DmSRS2, antioxidants were added to flies’ diet and we observed an amelioration in both phenotypes (Figure 8). The commercial antioxidant mix used (K-PAX; K-PAX Inc.) is a complex combination of compounds, and thus it is difficult to discuss the beneficial effect observed in this work. However, the significant palliative effect observed warrants a deeper analysis on the potential therapeutic effect of ROS inhibitors in diseases caused by mitochondrial malfunction.

In support of this conclusion it should be noted that, during the preparation of this manuscript, a fly model for ataxia with leukoencephalopathy caused by rearrangements on methionyl-tRNA synthetase 2 gene was published (21). The phenotype observed in our model closely coincide with the model from Bayat et al., with the same kind of mitochondrial morphology and respiratory defects, as well as a build-up in mitochondrial biogenesis and ROS accumulation, and a reduction in cell proliferation independent of apoptosis and cell growth events.

Our animal model is able to reproduce many traits that characterize mitochondrial disorders caused by mutations in the mitochondrial serylation apparatus. As example, an insertion in the mt-tRNA^Ser^ that cause sensorineural hearing loss, results in a reduction in serylation efficiency, a moderate mitochondrial dysfunction, morphological alterations and lactate elevation (65,66). Mutations in mt-tRNA^Ser^ related to Multisystem Disease with Cataracts (67) and deafness, retinal degeneration, myopathy and epilepsy (68) cause defects in mitochondrial function, abnormal mitochondrial morphology and proliferation, and those involved in MELAS/MERRF result in a group of features, such as pleomorphic mitochondria, increment in lactate, decrease in respiratory chain activity and increase in mitochondrial density (69), that coincide with the phenotypes observed in our model. On the other hand, similar symptoms have also been observed in patients with HUPRA syndrome (hyperuricemia, pulmonary hypertension, renal failure in infancy and alkalosis), which is caused by a mutation in the SRS2 gene (18).

The increasing number of pathogenic mutations found in mitochondrial aaRSs (15–23) justifies the generation of animal models for the study of these diseases and for the development of therapeutic strategies, among which treatment with antioxidant molecules should be considered.

## SUPPLEMENTARY DATA

Supplementary Data are available at NAR Online: Supplementary Figures 1–3 and Supplementary Methods.

## FUNDING

Spanish Ministry of Economy and Innovation [BIO2012-32200]; PhD fellowship from the Government of Catalonia [FI-DGR 2011 to D.P.]. Funding for open access charge: Spanish Ministry of Economy and Innovation [BIO2013-09776].

*Conflict of interest statement*. None declared.

## Supplementary Material

Supplementary Data

## References

[1] Ibba M, Söll D (2000). Aminoacyl-tRNA synthesis. Annu. Rev. Biochem..

[2] Ruiz-Pesini E, Lott MT, Procaccio V, Poole JC, Brandon MC, Mishmar D, Yi C, Kreuziger J, Baldi P, Wallace DC (2007). An enhanced MITOMAP with a global mtDNA mutational phylogeny. Nucleic Acids Res..

[3] Goto Y, Nonaka I, Horai S (1990). A mutation in the tRNA(Leu)(UUR) gene associated with the MELAS subgroup of mitochondrial encephalomyopathies. Nature.

[4] Kobayashi Y, Momoi MY, Tominaga K, Momoi T, Nihei K, Yanagisawa M, Kagawa Y, Ohta S (1990). A point mutation in the mitochondrial tRNA(Leu)(UUR) gene in MELAS (mitochondrial myopathy, encephalopathy, lactic acidosis and stroke-like episodes). Biochem. Biophys. Res. Commun..

[5] Shoffner JM, Lott MT, Lezza AM, Seibel P, Ballinger SW, Wallace DC (1990). Myoclonic epilepsy and ragged-red fiber disease (MERRF) is associated with a mitochondrial DNA tRNA(Lys) mutation. Cell.

[6] Reid FM, Vernham GA, Jacobs HT (1994). A novel mitochondrial point mutation in a maternal pedigree with sensorineural deafness. Hum. Mutat..

[7] Scheper GC, van der Knaap MS, Proud CG (2007). Translation matters: protein synthesis defects in inherited disease. Nat. Rev. Genet..

[8] Antonellis A, Ellsworth RE, Sambuughin N, Puls I, Abel A, Lee-Lin SQ, Jordanova A, Kremensky I, Christodoulou K, Middleton LT (2003). Glycyl tRNA synthetase mutations in Charcot-Marie-Tooth disease type 2D and distal spinal muscular atrophy type V. Am. J. Hum. Genet..

[9] Jordanova A, Irobi J, Thomas FP, Van Dijck P, Meerschaert K, Dewil M, Dierick I, Jacobs A, De Vriendt E, Guergueltcheva V (2006). Disrupted function and axonal distribution of mutant tyrosyl-tRNA synthetase in dominant intermediate Charcot-Marie-Tooth neuropathy. Nat. Genet..

[10] Latour P, Thauvin-Robinet C, Baudelet-Mery C, Soichot P, Cusin V, Faivre L, Locatelli MC, Mayencon M, Sarcey A, Broussolle E (2010). A major determinant for binding and aminoacylation of tRNA(Ala) in cytoplasmic Alanyl-tRNA synthetase is mutated in dominant axonal Charcot-Marie-Tooth disease. Am. J. Hum. Genet..

[11] McLaughlin HM, Sakaguchi R, Liu C, Igarashi T, Pehlivan D, Chu K, Iyer R, Cruz P, Cherukuri PF, Hansen NF (2010). Compound heterozygosity for loss-of-function lysyl-tRNA synthetase mutations in a patient with peripheral neuropathy. Am. J. Hum. Genet..

[12] He W, Zhang HM, Chong YE, Guo M, Marshall AG, Yang XL (2011). Dispersed disease-causing neomorphic mutations on a single protein promote the same localized conformational opening. Proc. Natl Acad. Sci. USA.

[13] Froelich CA, First EA (2011). Dominant Intermediate Charcot-Marie-Tooth disorder is not due to a catalytic defect in tyrosyl-tRNA synthetase. Biochemistry.

[14] Motley WW, Seburn KL, Nawaz MH, Miers KE, Cheng J, Antonellis A, Green ED, Talbot K, Yang XL, Fischbeck KH (2011). Charcot-Marie-Tooth-linked mutant GARS is toxic to peripheral neurons independent of wild-type GARS levels. PLoS Genet..

[15] Scheper GC, van der Klok T, van Andel RJ, van Berkel CG, Sissler M, Smet J, Muravina TI, Serkov SV, Uziel G, Bugiani M (2007). Mitochondrial aspartyl-tRNA synthetase deficiency causes leukoencephalopathy with brain stem and spinal cord involvement and lactate elevation. Nat. Genet..

[16] Edvardson S, Shaag A, Kolesnikova O, Gomori JM, Tarassov I, Einbinder T, Saada A, Elpeleg O (2007). Deleterious mutation in the mitochondrial arginyl-transfer RNA synthetase gene is associated with pontocerebellar hypoplasia. Am. J. Hum. Genet..

[17] Riley LG, Cooper S, Hickey P, Rudinger-Thirion J, McKenzie M, Compton A, Lim SC, Thorburn D, Ryan MT, Giege R (2010). Mutation of the mitochondrial tyrosyl-tRNA synthetase gene, YARS2, causes myopathy, lactic acidosis, and sideroblastic anemia—MLASA syndrome. Am. J. Hum. Genet..

[18] Belostotsky R, Ben-Shalom E, Rinat C, Becker-Cohen R, Feinstein S, Zeligson S, Segel R, Elpeleg O, Nassar S, Frishberg Y (2011). Mutations in the mitochondrial seryl-tRNA synthetase cause hyperuricemia, pulmonary hypertension, renal failure in infancy and alkalosis, HUPRA syndrome. Am. J. Hum. Genet..

[19] Pierce SB, Chisholm KM, Lynch ED, Lee MK, Walsh T, Opitz JM, Li W, Klevit RE, King MC (2011). Mutations in mitochondrial histidyl tRNA synthetase HARS2 cause ovarian dysgenesis and sensorineural hearing loss of Perrault syndrome. Proc. Natl Acad. Sci. USA.

[20] Götz A, Tyynismaa H, Euro L, Ellonen P, Hyotylainen T, Ojala T, Hamalainen RH, Tommiska J, Raivio T, Oresic M (2011). Exome sequencing identifies mitochondrial alanyl-tRNA synthetase mutations in infantile mitochondrial cardiomyopathy. Am. J. Hum. Genet..

[21] Bayat V, Thiffault I, Jaiswal M, Tetreault M, Donti T, Sasarman F, Bernard G, Demers-Lamarche J, Dicaire MJ, Mathieu J (2012). Mutations in the mitochondrial methionyl-tRNA synthetase cause a neurodegenerative phenotype in flies and a recessive ataxia (ARSAL) in humans. PLoS Biol..

[22] Steenweg ME, Ghezzi D, Haack T, Abbink TE, Martinelli D, van Berkel CG, Bley A, Diogo L, Grillo E, Te Water Naude J (2012). Leukoencephalopathy with thalamus and brainstem involvement and high lactate ‘LTBL' caused by EARS2 mutations. Brain.

[23] Elo JM, Yadavalli SS, Euro L, Isohanni P, Gotz A, Carroll CJ, Valanne L, Alkuraya FS, Uusimaa J, Paetau A (2012). Mitochondrial phenylalanyl-tRNA synthetase mutations underlie fatal infantile Alpers encephalopathy. Hum. Mol. Genet..

[24] Seburn KL, Nangle LA, Cox GA, Schimmel P, Burgess RW (2006). An active dominant mutation of glycyl-tRNA synthetase causes neuropathy in a Charcot-Marie-Tooth 2D mouse model. Neuron.

[25] Chihara T, Luginbuhl D, Luo L (2007). Cytoplasmic and mitochondrial protein translation in axonal and dendritic terminal arborization. Nat. Neurosci..

[26] Achilli F, Bros-Facer V, Williams HP, Banks GT, AlQatari M, Chia R, Tucci V, Groves M, Nickols CD, Seburn KL (2009). An ENU-induced mutation in mouse glycyl-tRNA synthetase (GARS) causes peripheral sensory and motor phenotypes creating a model of Charcot-Marie-Tooth type 2D peripheral neuropathy. Dis. Model. Mech..

[27] Storkebaum E, Leitao-Goncalves R, Godenschwege T, Nangle L, Mejia M, Bosmans I, Ooms T, Jacobs A, Van Dijck P, Yang XL (2009). Dominant mutations in the tyrosyl-tRNA synthetase gene recapitulate in *Drosophila* features of human Charcot-Marie-Tooth neuropathy. Proc. Natl Acad. Sci. USA.

[28] Butow RA, Henke RM, Moran JV, Belcher SM, Perlman PS (1996). Transformation of *Saccharomyces cerevisiae* mitochondria using the biolistic gun. Methods Enzymol..

[29] Bonnefoy N, Fox TD (2001). Genetic transformation of *Saccharomyces cerevisiae* mitochondria. Methods Cell Biol..

[30] Feuermann M, Francisci S, Rinaldi T, De Luca C, Rohou H, Frontali L, Bolotin-Fukuhara M (2003). The *yeast* counterparts of human ‘MELAS' mutations cause mitochondrial dysfunction that can be rescued by overexpression of the mitochondrial translation factor EF-Tu. EMBO Rep..

[31] King MP, Attardi G (1989). Human cells lacking mtDNA: repopulation with exogenous mitochondria by complementation. Science.

[32] Swerdlow RH (2007). Mitochondria in cybrids containing mtDNA from persons with mitochondriopathies. J. Neurosci. Res..

[33] Toivonen JM, O'Dell KM, Petit N, Irvine SC, Knight GK, Lehtonen M, Longmuir M, Luoto K, Touraille S, Wang Z (2001). Technical knockout, a *Drosophila* model of mitochondrial deafness. Genetics.

[34] Kolesnikova OA, Entelis NS, Jacquin-Becker C, Goltzene F, Chrzanowska-Lightowlers ZM, Lightowlers RN, Martin RP, Tarassov I (2004). Nuclear DNA-encoded tRNAs targeted into mitochondria can rescue a mitochondrial DNA mutation associated with the MERRF syndrome in cultured human cells. Hum. Mol. Genet..

[35] Mahata B, Mukherjee S, Mishra S, Bandyopadhyay A, Adhya S (2006). Functional delivery of a cytosolic tRNA into mutant mitochondria of human cells. Science.

[36] Karicheva OZ, Kolesnikova OA, Schirtz T, Vysokikh MY, Mager-Heckel AM, Lombes A, Boucheham A, Krasheninnikov IA, Martin RP, Entelis N (2011). Correction of the consequences of mitochondrial 3243A>G mutation in the MT-TL1 gene causing the MELAS syndrome by tRNA import into mitochondria. Nucleic Acids Res..

[37] Wang G, Shimada E, Zhang J, Hong JS, Smith GM, Teitell MA, Koehler CM (2012). Correcting human mitochondrial mutations with targeted RNA import. Proc. Natl Acad. Sci. USA.

[38] Park H, Davidson E, King MP (2008). Overexpressed mitochondrial leucyl-tRNA synthetase suppresses the A3243G mutation in the mitochondrial tRNA(Leu(UUR)) gene. RNA.

[39] Li R, Guan MX (2010). Human mitochondrial leucyl-tRNA synthetase corrects mitochondrial dysfunctions due to the tRNA^Leu^(UUR) A3243G mutation, associated with mitochondrial encephalomyopathy, lactic acidosis, and stroke-like symptoms and diabetes. Mol. Cell. Biol..

[40] Marchington DR, Barlow D, Poulton J (1999). Transmitochondrial mice carrying resistance to chloramphenicol on mitochondrial DNA: developing the first mouse model of mitochondrial DNA disease. Nat. Med..

[41] Sligh JE, Levy SE, Waymire KG, Allard P, Dillehay DL, Nusinowitz S, Heckenlively JR, MacGregor GR, Wallace DC (2000). Maternal germ-line transmission of mutant mtDNAs from embryonic stem cell-derived chimeric mice. Proc. Natl Acad. Sci. USA.

[42] Inoue K, Nakada K, Ogura A, Isobe K, Goto Y, Nonaka I, Hayashi JI (2000). Generation of mice with mitochondrial dysfunction by introducing mouse mtDNA carrying a deletion into zygotes. Nat. Genet..

[43] Li J, Zhou K, Meng X, Wu Q, Li S, Liu Y, Wang J (2008). Increased ROS generation and SOD activity in heteroplasmic tissues of transmitochondrial mice with A3243G mitochondrial DNA mutation. Genet. Mol. Res..

[44] Guitart T, Leon Bernardo T, Sagales J, Stratmann T, Bernues J, Ribas de Pouplana L (2010). New aminoacyl-tRNA synthetase-like protein in insecta with an essential mitochondrial function. J. Biol. Chem..

[45] Lee YS, Carthew RW (2003). Making a better RNAi vector for *Drosophila*: use of intron spacers. Methods.

[46] Rubin GM, Spradling AC (1982). Genetic transformation of *Drosophila* with transposable element vectors. Science.

[47] Dietzl G, Chen D, Schnorrer F, Su KC, Barinova Y, Fellner M, Gasser B, Kinsey K, Oppel S, Scheiblauer S (2007). A genome-wide transgenic RNAi library for conditional gene inactivation in *Drosophila*. Nature.

[48] Brand AH, Perrimon N (1993). Targeted gene expression as a means of altering cell fates and generating dominant phenotypes. Development.

[49] Saini N, Georgiev O, Schaffner W (2011). The parkin mutant phenotype in the fly is largely rescued by metal-responsive transcription factor (MTF-1). Mol. Cell. Biol..

[50] Walker SE, Fredrick K (2008). Preparation and evaluation of acylated tRNAs. Methods.

[51] Wilhelm ML, Baranowski W, Keith G, Wilhelm FX (1992). Rapid transfer of small RNAs from a polyacrylamide gel onto a nylon membrane using a gel dryer. Nucleic Acids Res..

[52] Abramoff MD, Magelhaes PJ, Ram SJ (2004). Image processing with imageJ. Biophotonics Int..

[53] Gutmann I, Wahlefel AW (1974). Methods of Enzymatic Analysis (Bergmeyer H. U.).

[54] Zhu A, Romero R, Petty HR (2009). An enzymatic fluorimetric assay for glucose-6-phosphate: application in an in vitro Warburg-like effect. Anal. Biochem..

[55] Boushel R, Gnaiger E, Schjerling P, Skovbro M, Kraunsoe R, Dela F (2007). Patients with type 2 diabetes have normal mitochondrial function in skeletal muscle. Diabetologia.

[56] Owusu-Ansah E, Yavari A, Banerjee U (2008). A protocol for in vivo detection of reactive oxygen species. Nat. Protoc..

[57] Duffy JB (2002). GAL4 system in *Drosophila*: a fly geneticist's Swiss army knife. Genesis.

[58] Phillips RG, Roberts IJ, Ingham PW, Whittle JR (1990). The *Drosophila* segment polarity gene *patched* is involved in a position-signalling mechanism in imaginal discs. Development.

[59] Ogasawara E, Nakada K, Hayashi J (2010). Lactic acidemia in the pathogenesis of mice carrying mitochondrial DNA with a deletion. Hum. Mol. Genet..

[60] Gnaiger E (2011). MitoPathways: Respiratory States ant Coupling Control Ratios.

[61] Mita S, Tokunaga M, Kumamoto T, Uchino M, Nonaka I, Ando M (1995). Mitochondrial DNA mutation and muscle pathology in mitochondrial myopathy, encephalopathy, lactic acidosis, and strokelike episodes. Muscle Nerve.

[62] Oldfors A, Holme E, Tulinius M, Larsson NG (1995). Tissue distribution and disease manifestations of the tRNA(Lys) A—>G(8344) mitochondrial DNA mutation in a case of myoclonus epilepsy and ragged red fibres. Acta Neuropathol..

[63] Melone MA, Tessa A, Petrini S, Lus G, Sampaolo S, di Fede G, Santorelli FM, Cotrufo R (2004). Revelation of a new mitochondrial DNA mutation (G12147A) in a MELAS/MERRF phenotype. Arch. Neurol..

[64] Lee HC, Wei YH (2005). Mitochondrial biogenesis and mitochondrial DNA maintenance of mammalian cells under oxidative stress. Int. J. Biochem. Cell Biol..

[65] Toompuu M, Yasukawa T, Suzuki T, Hakkinen T, Spelbrink JN, Watanabe K, Jacobs HT (2002). The 7472insC mitochondrial DNA mutation impairs the synthesis and extent of aminoacylation of tRNASer(UCN) but not its structure or rate of turnover. J. Biol. Chem..

[66] Cardaioli E, Da Pozzo P, Cerase A, Sicurelli F, Malandrini A, De Stefano N, Stromillo ML, Battisti C, Dotti MT, Federico A (2006). Rapidly progressive neurodegeneration in a case with the 7472insC mutation and the A7472C polymorphism in the mtDNA tRNA^Ser^(UCN) gene. Neuromuscul. Disord..

[67] Schrier SA, Wong LJ, Place E, Ji JQ, Pierce EA, Golden J, Santi M, Anninger W, Falk MJ (2012). Mitochondrial tRNA-serine (AGY) m.C12264T mutation causes severe multisystem disease with cataracts. Discov. Med..

[68] Tuppen HA, Naess K, Kennaway NG, Al-Dosary M, Lesko N, Yarham JW, Bruhn H, Wibom R, Nennesmo I, Weleber RG (2012). Mutations in the mitochondrial tRNA(Ser(AGY)) gene are associated with deafness, retinal degeneration, myopathy and epilepsy. Eur. J. Hum. Genet..

[69] Wong LJ, Yim D, Bai RK, Kwon H, Vacek MM, Zane J, Hoppel CL, Kerr DS (2006). A novel mutation in the mitochondrial tRNA(Ser(AGY)) gene associated with mitochondrial myopathy, encephalopathy, and complex I deficiency. J. Med. Genet..

