# Supplementary material for: Human mitochondrial disease-like symptoms caused by a reduced tRNA aminoacylation activity in flies
Source: Nucleic Acids Res. 2013 May 15;41(13):6595–608. doi: 10.1093/nar/gkt402 (PMC3711456; doi:10.1093/nar/gkt402)
Supplement: Supplementary Data [file supp_41_13_6595__index.html]

Human mitochondrial disease-like symptoms caused by a reduced tRNA aminoacylation activity in flies — Human mitochondrial disease-like symptoms caused by a reduced tRNA aminoacylation activity in flies — Supplementary Data 

# Human mitochondrial disease-like symptoms caused by a reduced tRNA aminoacylation activity in flies

## Supplementary Data

files

**Files in this Data Supplement:**

- Supplementary Data - pdf file
