# Supplementary material for: Human mitochondrial disease-like symptoms caused by a reduced tRNA aminoacylation activity in flies
Source: Nucleic Acids Res. 2013 May 15;41(13):6595–608. doi: 10.1093/nar/gkt402 (PMC3711456; doi:10.1093/nar/gkt402)
Supplement: Supplementary Data [file supp_gkt402_nar-03375-v-2012-File010.pdf]

SUPPLEMENTARY MATERIAL AND METHODS:

Quantitative real-time PCR. Total RNA was extracted from third instar larvae with TRIzol (Invitrogen), digested with DNase I and cleaned with the RNeasy MinElute Cleanup kit (Qiagen). 1 μg of total RNA was retrotranscribed into cDNA using oligo(dT) primers to perform quantitative real-time PCRs by means of *Power* SYBR Green and a StepOnePlus Real-time PCR System (Applied Biosystems). cDNA templates were amplified with a pair of primers designed with the Primer Express® software (Applied Biosystems) to detect the DmSRS1 cDNA (5'GACCTCACAGAGATTGTAGCC3' and 5'CTCTAGCGACTTCTGGTTCTC3'), the SLIMP cDNA (5'GGCGATAAAGCGAACGAAAAC3' and 5'AAAAATTGCCGCTCTCCAAA3') and another to detect the Rp49 cDNA, used as endogenous control (5'TGCCCACCGGATTCAAGA3' and 5'AAACGCGGTTCTGCATGAG3'). Standard curves were calculated for both primer pairs to ensure a high efficiency level. 20 μL reactions were prepared following the manufacturer's instructions, using ROX as reference dye and the following conditions: 50 ºC for 2 min; 95 ºC for 10 min; 40 cycles (95 ºC for 15 s; 60 for 1 min). Fold expression changes were calculated using the $2^{-\Delta\Delta CT}$ method. The value obtained for control larvae is represented as 1 and the other values are represented relative to it.

Analysis of aminoacylated and deacylated mt-tRNA. Total RNA was extracted with TRIzol (Invitrogen) from w^{1118} third instar larvae. RNA was deacylated by incubating 1 h at 37 ºC in 1 M Tris pH 8.0 and 1 mM EDTA. 30 μg of deacylated or untreated RNA were electrophoresed on high resolution acid gels and analysed by Northern blot using the following radiolabeled probes: 5'TGGTCATTAGAAGTAAGTGCTAATTTAC3' for $mt\text{-}tRNA^{Lys}$ (CUU) and 5'TGGAGAAATATAAATGGAATTTAACC3' for $mt\text{-}tRNA^{Ser}$ (GCU). Signals were digitalized using a PhosphorImager™ from a gel exposed storage phosphor screen.

Western blot. Third-instar larvae ($RNAi_{DmSRS2}$ strain 1-*dcr2*, $RNAi_{DmSRS2}$ strain 23003 and w^{1118}) were collected, frozen and homogenized using ice-cold lysis buffer (1% NP-40; 150mM NaCl; 50mM Tris pH 8; 5mM EDTA) supplemented just before use with Complete™ EDTA-free protease inhibitor cocktail (Roche). The homogenates were centrifuged at 16,000 g for 30 minutes at 4 °C. Equal amounts of protein lysates were aliquoted and mixed with an equal volume of 2x loading buffer (125 mM TrisHCl, pH 7.2; 4% SDS; 100mM DTT; 20% glycerol; 0.01% bromophenol blue). Samples were boiled for 5 min, resolved on 10% SDS-PAGE gel and transferred to Immobilon™-PVDF membrane. Blots were blocked in 5% milk and incubated with rabbit anti-MT-ND1 antibody at 1:500 (Abcam #ab74257) and mouse anti-MT-CO2 antibody at 1:500 (Invitrogen #A6404) at 4 °C overnight. A polyclonal serum against

Drosophila β-ATPase 1:2000 (gift from Dr. Rafael Garesse, IIB-UAM) was used as mitochondrial marker and loading control. Anti-β-tubulin antibody 1:2000 was used as a loading control (Millipore, MAB3408). HRP-conjugated secondary antibodies were used and chemiluminescence was detected by ECL Advance Western Blotting detection kit (Amersham). Signal was digitalized and quantified by ImageJ software.

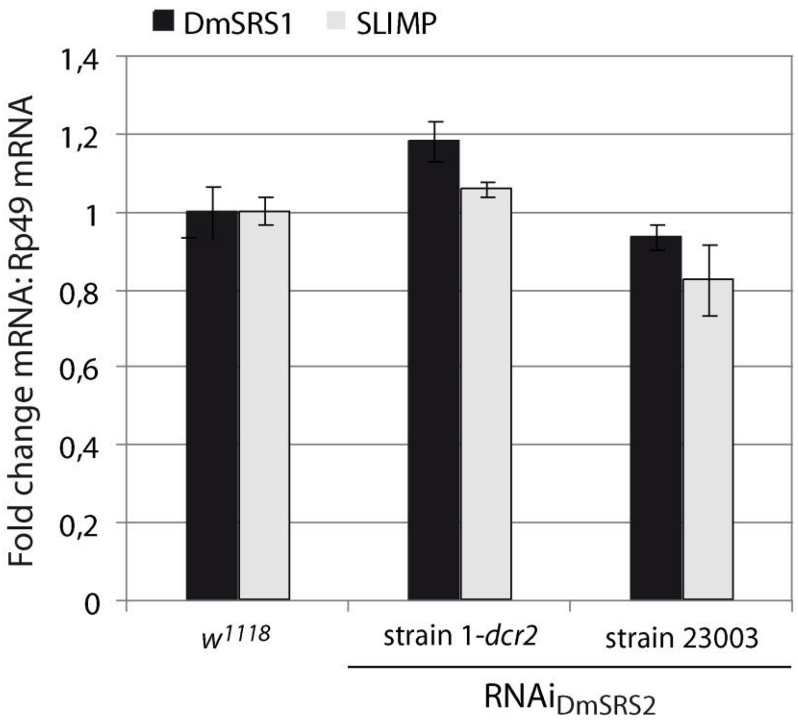

Supplementary Figure 1. DmSRS2 knock-down specificity. As a control for the specificity of the silencing of DmSRS2, the mRNA levels of the cytosolic SRS (DmSRS1) and the DmSRS2 paralogous SLIMP were quantified by real-time PCR in control larvae (w^{1118}) and $\mathrm{RNAi}_{\mathrm{DmSRS2}}$ larvae from strains 1-*dcr2* and 23003 crossed with actin5C-GAL4 driver at 29 ºC and 25 ºC, respectively. DmSRS1and SLIMP mRNA levels were normalized using Rp49 mRNA as reference. Graph gives average with S.E.M. from three independent experiments. Statistical significance was calculated by Student *t*-test and no significant difference was found. The mRNA level in control larvae is established as 1 and the other values are relative to this.

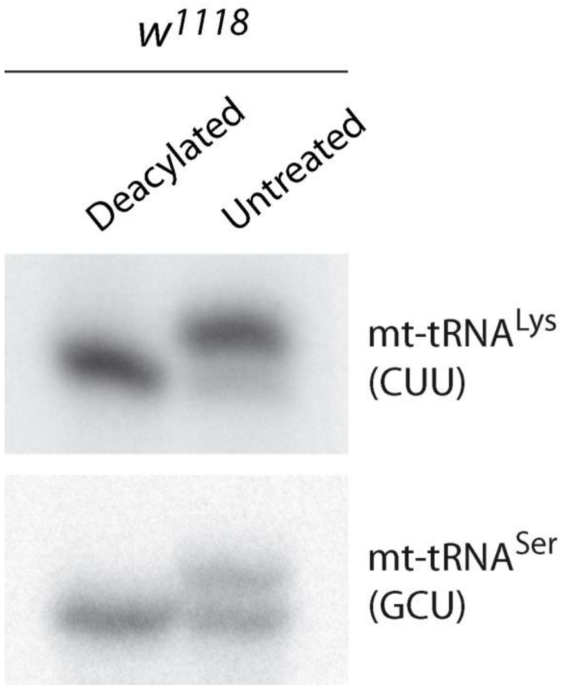

Supplementary Figure 2. Aminoacylation levels of mt-tRNAs. 30μg of deacylated or untreated RNA from *w*^{1118} larvae were loaded into high resolution acid gels. mt-tRNA^{Lys} (CUU) and mt-tRNA^{Ser} (GCU) were specifically detected by Northern blot. While mt-tRNA^{Lys} (CUU) is aminoacylated at around the 100 %, the basal levels of aminoacylation for mt-tRNA^{Ser} (GCU) are around 50-60 %.

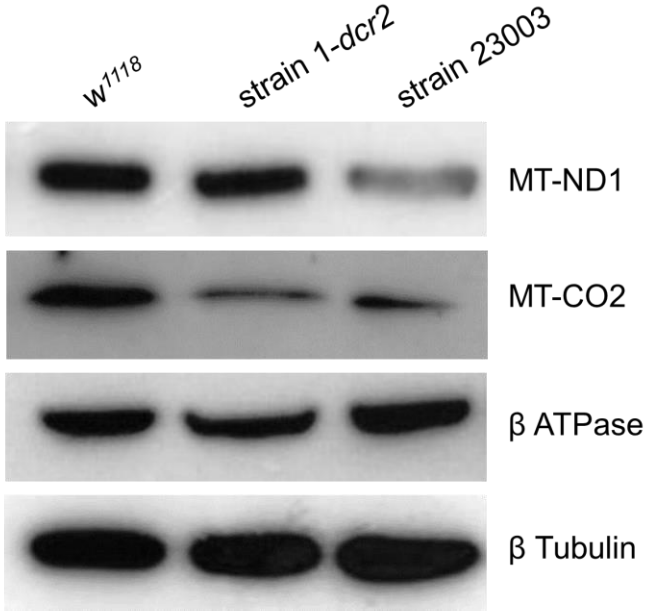

Supplementary Figure 3. DmSRS2 knock-down affects mitochondrial translation. The antibody anti-MT-ND1 detects endogenous levels of total MT-ND1 protein, a mitochondrial encoded core subunit of the mitochondrial membrane respiratory chain NADH dehydrogenase (Complex I). The antibody anti-MT-CO2 detects endogenous levels of subunit 2 of the cytochrome c oxidase (Complex IV). The figure shows the decrease of MT-ND1 and MT-CO2 proteins in mutant larvae emerging from the crosses between actin5C-GAL4 and $\text{RNAi}_{\text{DmSRS2}}$ strain 1-*dcr2* at 29ºC (MT-ND1: 5.2% decrease; MT-CO2: 43,6% decrease) or strain 23003 at 25 ºC (MT-ND1: 48,7% decrease; MT-CO2: 51,3% decrease), compared to that from the control w^{1118}. β-ATPase and β-tubulin were used as mitochondrial and total loading control, respectively.
